# Benign Etiology for High-Risk Intraductal Papillary Mucinous Neoplasm: A Case Report and Literature Review

**DOI:** 10.7759/cureus.62054

**Published:** 2024-06-10

**Authors:** Ryan J Kramer, Chanjuan Shi, Dimitrios Moris, Peter J Allen

**Affiliations:** 1 School of Medicine, Duke University School of Medicine, Durham, USA; 2 Department of Pathology, Duke University Medical Center, Durham, USA; 3 Department of Surgery, Duke University Medical Center, Durham, USA; 4 Department of Surgical Oncology, Duke University Medical Center, Durham, USA

**Keywords:** pancreatic cyst management, ca19-9, whipple procedure, pancreatic cancer, intraductal papillary mucinous neoplasm (ipmn)

## Abstract

Intraductal papillary mucinous neoplasms are relatively common and entail a variable risk of malignant potential. The Fukuoka guidelines present criteria for the risk of malignant transformation and are used for risk stratification and treatment decision-making. However, these guidelines entail some fallibility with limited sensitivity and specificity. In this case, we present an individual who had many of the hallmarks of malignant transformation but was found to have no evidence of malignancy or high-grade dysplasia. We discuss the suspected etiology of this individual’s condition and how it might arise in others, as well as a brief review of the literature on risk factors in intraductal papillary mucinous neoplasms.

## Introduction

Cystic pancreatic lesions represent a heterogeneous entity with a range of malignant potential. Lesions such as serous cystadenomas and pancreatic fluid collections have no risk of malignancy [1]. Contrariwise, solid pseudopapillary neoplasms, cystic pancreatic endocrine neoplasms, mucinous cystic neoplasms, and intraductal papillary mucinous neoplasms (IPMNs) all entail some risk of malignant transformation [1]. As such, substantial effort has been spent on developing risk-stratification algorithms to identify individuals at risk of malignancy for surgical resection.

The Fukuoka guidelines have been established to identify high-risk features of IPMN disease and guide resection decisions. An IPMN is Fukuoka-positive and should be resected if any of the following occur: obstructive jaundice, contrast-enhancing mural nodules greater than 5 mm, and/or dilatation of the main pancreatic duct greater than 10 mm [2]. However, IPMNs that are not Fukuoka-positive may still warrant resection in certain patients given sufficient "worrisome features," including cyst location, radiologic appearance, growth, and tumor markers [2]. We present a case of an 84-year-old male with a growing, high-risk IPMN and elevated serum carbohydrate antigen (CA)19-9 (peak value of 660 U/mL) who was brought to the OR for resection and aborted due to vascular adhesions. However, intraoperative biopsy pathology demonstrated that this cyst was benign without even high-grade dysplasia, and he remains alive today.

Thus, this case represents a benign process recapitulating the stigmata of malignancy. The high-risk features concerning malignancy were, in actuality, most likely secondary to an iatrogenic intra-cystic bleed during the endoscopic ultrasound and fine needle aspiration. To our knowledge, this is the first documentation of such a case. In addition to this case, we include a brief review of the literature on IPMN risk stratification and decision-making guidelines.

## Case presentation

**Figure 1:**
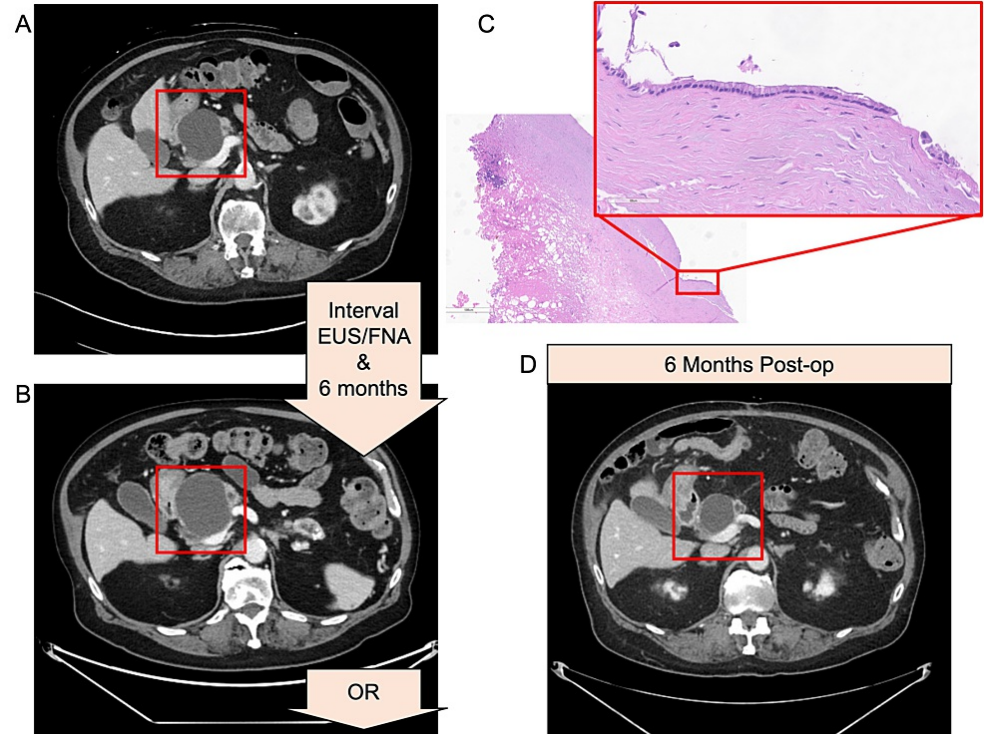
Radiographic and pathologic evidence. (A) The pancreas protocol CT that led to a referral to surgical oncology. (B) The six-month follow-up pancreas protocol CT demonstrated cyst growth. In these six months, the patient also had an EUS/FNA. (C) Operative cyst wall biopsy demonstrated a fibrotic cystic wall with focal residual epithelial lining. A representative high-power field shows mucinous epithelium with no high-grade dysplasia. (D) The six-month post-operative follow-up pancreas protocol CT demonstrated cyst shrinkage. EUS: endoscopic ultrasound, FNA: fine needle aspiration.

An 84-year-old male presented to the surgical oncology clinic for evaluation of an enlarging branch-duct IPMN located in the pancreatic head. This lesion was discovered incidentally six years prior, when it was 3.5 cm in diameter. He did not undergo routine surveillance; however, two years prior to presentation to surgical oncology, he underwent an ED evaluation for constipation, and on CT imaging (Figure 1A), this lesion was found to have grown to 5.6 cm in diameter with associated 5 mm main pancreatic ductal dilatation (the normal main pancreatic ductal diameter per Fukuoka guidelines is <5 mm [2]).

On presentation to surgical oncology, his CA19-9 was 54 U/mL and carcinoembryonic antigen (CEA) was 3.9 ng/mL (Table 1). An abdominal MRI was ordered, which re-demonstrated the 5.5 cm, non-septated cyst without a solid component but with diffuse pancreatic duct dilatation to 6 mm. He then underwent an endoscopic ultrasound (EUS) with fine needle aspiration (FNA). EUS reiterated a 5.8 cm cyst in the pancreatic head without septation. Adjacent to the cyst was a 2.3 cm hypoechoic mass with poorly defined borders, which was also aspirated four times. FNA results demonstrated “fragments of bland mucinous epithelium and macrophages.” The cyst fluid labs demonstrated cyst fluid amylase of 3,404 U/L and cyst fluid CEA of 2,056 ng/mL (Table 1). These results supported the presence of IPMN, interval growth of more than 2 cm, 6 mm ductal dilatation, and elevated serum tumor markers; it was considered to be high-risk, and resection was recommended. At that time, the patient declined the recommendation for resection and chose radiographic surveillance.

**Table 1:** Preoperative laboratory values. CEA: carcinoembryonic antigen.

Time interval	Lab	Value	Normal range
Baseline	Serum CA19-9 (U/mL)	54	≤35
Baseline	Serum CEA (ng/mL)	3.9	≤2.5
Baseline	Cyst amylase (U/L)	3,404	N/A
Baseline	Cyst CEA (ng/mL)	2,056	≤200
Six-month follow-up	Serum CA19-9 (U/mL)	660	≤35

On follow-up imaging six months later, the cyst had grown to 6.5 cm with a new area of cyst complexity and mural thickening on CT (Figure 1B). Ductal dilatation had increased to 7 mm at the tail, with mild intrahepatic biliary ductal dilatation and common bile duct effacement at the level of the cyst. The patient’s serum CA19-9 had increased to 660 U/mL, a greater than tenfold increase over six months (Table 1). Consequently, the decision was made to proceed with resection due to concern for malignant transformation within the cyst.

The procedure was performed two weeks later. Intraoperatively, a midline laparotomy was performed, and a planned Whipple procedure was initiated. The cyst was anatomically unresectable due to significant peri-pancreatic inflammation and adhesions to the superior mesenteric vein, portal vein confluence, and hepatic artery. Thus, the Whipple was aborted. Three biopsies of the cystic wall were taken to achieve tissue diagnosis, and the cyst was drained to reduce compression. Intraoperatively, the cyst contents were noted to be bloody. The patient recovered well postoperatively.

Intraoperative frozen sections of the three cyst wall biopsies were negative for malignancy. This was later confirmed on final pathology (Figure 1C), which read two biopsies as “Benign fibroadipose tissues and nerves. Negative for malignancy” and the third biopsy as “Fragment of the mucinous epithelial-lined cyst. Negative for invasive carcinoma.” Tumor markers were reordered: postoperative day two CA19-9 was 326 U/mL (an over fifty percent decrease from preoperatively) and CEA was 3.6 ng/mL, the patient’s lowest recorded value. His CA19-9 continued to decrease over the following months (217 U/mL at one month postoperatively and 205 U/mL at two months postoperatively). At his postoperative six-month re-imaging (Figure 1D), the patient’s cyst measured 3.6 cm with a resolution of mural thickening and his CA19-9 was 118 U/mL (Table 2).

**Table 2:** Postoperative laboratory values. CEA: carcinoembryonic antigen.

Postoperative interval	Lab	Value	Normal range
Day two	Serum CA19-9 (U/mL)	326	≤35
Day two	Serum CEA (ng/mL)	3.6	≤2.5
Month one	Serum CA19-9 (U/mL)	217	≤35
Month two	Serum CA19-9 (U/mL)	205	≤35
Month six	Serum CA19-9 (U/mL)	118	≤35

This patient represented an individual with the stigmata of high-risk IPMN concerning its transformation into pancreatic adenocarcinoma. Specifically, from his initial consult with surgical oncology to the six-month follow-up surveillance visit, his cyst had grown 1 cm, new mural thickening was observed, pancreatic ductal dilatation had increased, new intrahepatic biliary ductal dilatation was observed secondary to common bile duct compression, and his CA19-9 had increased by 12-fold. However, he had no malignancy on any biopsy intraoperatively and demonstrated improvement postoperatively on both CT and CA19-9, incompatible with pancreatic adenocarcinoma.

We hypothesize that the cyst bled into itself following the EUS/FNA. This is supported by the fact that the cyst fluid during the EUS/FNA was clear and mucinous, and yet during the attempted Whipple, the cyst fluid was grossly bloody. During the FNA, six passes were taken with the needle, one of which may have induced bleeding into the cyst. The cyst bleeding into itself likely generated the rapid growth of the cyst. The cyst growth, in turn, compressed adjacent ductal tissue, thereby elevating serum CA19-9 [3,4]. Thus, this patient presents a case concerning IPMN progression into malignancy necessitating intervention; however, this patient's case was most likely secondary to iatrogenic bleeding into the IPMN.

## Discussion

In the general population, the incidence of IPMN has been estimated to be 1.2-2.6% on CT and 2.4-49.1% on MRI [1,5]. The risk of progression to invasive disease has been estimated to be 19%-30% in branch duct-IPMN and 40%-60% in main duct-IPMN [6]; as such, substantial effort has been made to identify high-risk features that would indicate resection. The revised 2017 Fukuoka guidelines put forth several criteria. Fukuoka-positive IPMNs include those presenting with obstructive jaundice, possessing contrast-enhancing mural nodules >5 mm, and dilatation of the pancreatic duct >10 mm [2]. There are also several “worrisome features” put forth by the guidelines, including cystic lesions >3 cm, thickened cyst walls that may take up contrast, dilatation of the pancreatic duct 5-10 mm, change in pancreatic duct caliber and distal atrophy of the pancreas, lymphadenopathy, elevation in serum CA19-9, cyst growth >5 mm over two years, and/or clinical pancreatitis [2]. A meta-analysis with 983 patients with branch duct-IPMN demonstrated a sensitivity of 83% and specificity of 53% for the Fukuoka criteria [7]. Indeed, this case is relevant as it meets the criteria for resection but was nonetheless not malignant, nor even high-grade.

In this case, the elevation in CA19-9 was a notable factor that was concerning for malignancy. In the setting of substantial cyst growth, this was a reasonable interpretation. However, CA19-9 is a tumor-associated but not tumor-specific glycoprotein synthesized in normal pancreaticobiliary ductal epithelium [8] and can be elevated in a variety of settings ranging from malignancy to benign inflammatory conditions such as cholangitis or pancreatitis [4,8]. Used as a tumor marker, CA19-9 elevation has a sensitivity of 79%-95% and a specificity of 82%-91% for pancreatic adenocarcinoma among symptomatic patients [4]. However, for asymptomatic all-comers, the positive predictive value of CA19-9 elevation is only 0.9%, justifying the omission of CA19-9 screening [8]. The poor positive predictive value is primarily owed to the rarity of pancreatic adenocarcinoma; in IPMN, where cancer is more prevalent, one cohort found sensitivity and specificity to be 74.0%-90.7% and 51.1%-85.9%, respectively [9,10]. Of note, this implies approximately 15-50% of those with IPMN and elevated CA19-9 do not have pancreatic adenocarcinoma, consistent with this patient.

## Conclusions

Retrospectively, taking this patient to the OR for resection was reasonable given the growth and tumor markers, even despite the negative FNA. Thus, surgeons must recognize that CA19-9 can be elevated in the setting of benign pancreaticobiliary ductal damage, so interval growth of a cyst due to other causes (in this case, iatrogenic bleeding) is capable of engendering elevation. In particular, CA19-9 has only moderate specificity among those with IPMN. Thus, if a patient with IPMN has no cytologic or pathologic evidence of malignancy, other etiologies, such as this one, might be considered.

The patient continues to do well and has no abdominal complaints now 10 months following exploration. He will continue to be seen every six months for surveillance labs and CT.
